# Executive function abnormalities in pathological gamblers

**DOI:** 10.1186/1745-0179-4-7

**Published:** 2008-03-27

**Authors:** Donatella Marazziti, Mario Catena Dell'Osso, Ciro Conversano, Giorgio Consoli, Laura Vivarelli, Francesco Mungai, Elena Di Nasso, Francesca Golia

**Affiliations:** 1 Dipartimento di Psichiatria, Neurobiologia, Farmacologia e Biotecnologie, University of Pisa, Italy

## Abstract

**Background:**

Pathological gambling (PG) is an impulse control disorder characterized by persistent and maladaptive gambling behaviors with disruptive consequences for familial, occupational and social functions. The pathophysiology of PG is still unclear, but it is hypothesized that it might include environmental factors coupled with a genetic vulnerability and dysfunctions of different neurotransmitters and selected brain areas. Our study aimed to evaluate a group of patients suffering from PG by means of some neuropsychological tests in order to explore the brain areas related to the disorder.

**Methods:**

Twenty outpatients (15 men, 5 women), with a diagnosis of PG according to DSM-IV criteria, were included in the study and evaluated with a battery of neuropsychological tests: the Wisconsin Card Sorting Test (WCST), the Wechsler Memory Scale revised (WMS-R) and the Verbal Associative Fluency Test (FAS). The results obtained in the patients were compared with normative values of matched healthy control subjects.

**Results:**

The PG patients showed alterations at the WCST only, in particular they had a great difficulty in finding alternative methods of problem-solving and showed a decrease, rather than an increase, in efficiency, as they progressed through the consecutive phases of the test. The mean scores of the other tests were within the normal range.

**Conclusion:**

Our findings showed that patients affected by PG, in spite of normal intellectual, linguistic and visual-spatial abilities, had abnormalities emerging from the WCST, in particular they could not learn from their mistakes and look for alternative solutions. Our results would seem to confirm an altered functioning of the prefrontal areas which might provoke a sort of cognitive "rigidity" that might predispose to the development of impulsive and/or compulsive behaviors, such as those typical of PG.

## Background

In 1980, with the publication of the third edition of the Diagnostic and Statistical Manual of Mental Disorders (DSM-III) [1], the problem of excessive gambling was officially recognized as a psychiatric diagnosis. In DSM-IV-R [2] pathological gambling (PG) is defined as an impulse control disorder, characterized by persistent and maladaptive gambling behaviours, with disruptive consequences for familial, occupational and social functions. Epidemiological studies suggest that men, adolescents, ethnic minorities and patients with other psychiatric disorders are at a higher risk of suffering from PG [3], although more recent epidemiological data indicate that the disorder is progressing rapidly also amongst women and teen-agers [4,5]. Other risk factors include early exposure to gambling opportunities, such as those offered by the internet, cognitive deficits and relatives with PG or alcohol dependence [6-9].

Several studies have documented that PG patients are often affected by other psychiatric disorders, in particular depression, alcoholism or drug abuse (DA) [10-13]; the comorbid disorders are explained either as secondary to PG or primarily responsible for PG onset. Furthermore, PG is interpreted as belonging to the group of impulsive, compulsive, addictive behaviours which may share common genetic and/or etiological mechanisms [14].

Although the pathophysiology of PG is unclear, probably it represents the result of the interplay between individual and environmental factors. Different hypotheses have been put forward: a genetic vulnerability mainly involving dopamine receptors, biochemical dysfunctions at the level of the serotonin and dopamine systems and/or alterations of different brain areas.

Patients with ventromedial prefrontal cortex abnormalities show a peculiar impairment in decision making, as assessed by the gambling task, a specific test that explores the ability to balance immediate rewards against long-term consequences [15-17]. PG patients, as well as patients suffering from obsessive-compulsive disorder (OCD) or cocaine, opiate or alcohol abuse, present similar alterations when presented with the gambling task: these subjects continue to make choices according to an immediate reward, despite being fully aware of the long-term negative consequences [18,19]: taken together, these findings would suggest that all these heterogeneous patients may share a common dysfunction at the level of the ventromedial prefrontal cortex. In line with this hypothesis, a recent study using a comprehensive neuropsychological battery measuring executive functions, demonstrated that PG and alcohol-dependent patients showed a reduction of executive functioning performance on inhibition, time estimation, cognitive flexibility and planning tasks [20]. Moreover, neurocognitive indicators of decision-making and disinhibition, such as the Card Playing Task and Stop Signal Reaction Time respectively, seem to be powerful predictors of relapse in PG [21]. The impairement of decision-making observed in PG might be explained by the inability to inhibit irrelevant information: in a recent study, the performances on the reverse Stroop task, which highly discriminates the ability to inhibit interferences, were significantly impaired in PG patients than in healthy subjects [22].

PG has been associated to impulsivity and attention deficit: PG patients were found to perform significantly worse than control subjects on attention measures and showed more childhood behaviors related to attention deficits [23]. More recently, neuropsychological measures of impulsivity, such as the reaction time and number of errors at Go/No-go tasks, as well as the scores at the Barratt Impulsiveness Scale, were higher in PG patients than healthy control subjects [24], while highlighting the importance of this dimension in the clinical picture of PG.

Given the paucity of information in this field, our study aimed to evaluate the possible involvement of some brain areas in PG by means of a battery of neuropsychological tests, in particular the Wisconsin Card Sorting Test (WCST) [25], the Wechsler Memory Scale revised (WMS-R) [26] and the Verbal Associative Fluency Test (FAS) [27,28].

## Methods

Twenty outpatients (15 men, 5 women, mean age: 26 ± 4 years) with a diagnosis of PG according to the DSM-IVR criteria [2] were recruited at the Dipartimento di Psichiatria, Neurobiologia, Farmacologia e Biotecnologie at Pisa University, in the Psychiatry Section. None suffered from any severe physical illness nor had ever taken psychotropic drugs, except for two patients who had occasionally taken benzodiazepine for difficulty with sleeping. The mean score of PG at the Yale Brown for Obsessive Compulsive Disorder modified for PG (Y-BOCS-PG) [29] was 30 ± 2. The age of onset of the disorder (mean ± SD) was 17 ± 2 years.

Seven patients were suffering also from bipolar disorder (BD) of type II, 5 from OCD, 5 from DA (cocaine and cannabis) and 3 from alcohol abuse. Five patients had two comorbid disorders: 4 BD patients were suffering also from DA and 1 OCD patients from alcohol dependence.

The patients were compared with 20 matched healthy subjects (15 men, 5 women, mean age: 25 ± 5), who were selected from amongst a pool of 500 control individuals undergoing the same battery of neuropsychological tests. Both patients and control subjects were all right-handed.

### Neuropsychological Tests

Neuropsychological tests were administered by a psychologist (CC) in a relaxed setting, all together and following the same sequence: WCST, WMS-R and finally FAS. No subject reported any difficulty in completing the tests in about 30–40 minutes.

The WCST assesses abstract ability, namely the ability to shift cognitive strategies in response to changing environmental conditions, thereby assessing the kind of executive functioning which involves strategic planning, organized searching and the ability to use environmental feedback to modify cognitive sets. In this test, the subjects are required to take one card at a time from a pack and then place it below one of 4 different top cards, previously laid out on the table by the interviewer. They must pair off the cards according to categories of colour, shape or the number of stimuli reproduced on the card. The subjects have to discover the right strategy to follow in pairing off the stimulus cards with the top cards, making use of the feedback received from the interviewer. After each card has been put on the table, feedback is given. After 10 cards have been selected correctly in one category, the interviewer changes the category without informing the subject and from that moment on, answers which would have been correct for the previous category are considered wrong. The subjects have to change the principles according to which they pair off the cards in order to discover the new category chosen by the interviewer. The test lasts until 6 categories have been correctly identified, with no time limit.

The WMS-R includes verbal and visual learning tests by means of association and matching, memory of excerpts, drawings and the identification of visual spatial information previously presented. Memory is assessed in terms of both immediate and delayed recall. The WMS-R provides global values for general, verbal and visual short- and long-term memory, as well as for attention and concentration. Specific functionality is evaluated in the verbal areas of logical memory and associative learning with verbal pairing (immediate and delayed) along with the visual areas of memory of image, learning associated with visual stimuli (immediate and delayed) and visual reproduction of pictures (immediate and delayed), as well as the areas of mental concentration, numeric span and visual memory. Comparison of the index of attention/concentration with the general index of memory on the WMS-R provides a means of distinguishing between disorders of concentration and those of memory.

FAS is a test which evaluates the verbal fluency for phonological stimuli that is the ability to produce fluent and spontaneous language without unnecessary pauses or being unable to find more appropriate words. Verbal fluency is assessed typically by noting the number of words which an individual manages to pronounce with reference to a specific category (e.g., types of animals or words which begin with a specific letter). The patient is asked to think of the greatest number of words (excluding personal names and geographical places) beginning with a specific letter of the alphabet, until the interviewer interrupts the test. In the space of a minute for each of the three letters F, A, and S, subjects with a high-school certificate typically produce a total of 30–50 words, maintaining a steady flow for a whole minute. An inability to list 12 or more words for each letter indicates reduced verbal fluency.

### Statistical analysis

Parametric and non-parametric data of the two groups were compared by means of the Student t-test or chi-square analysis, respectively, all with personal computer programs, using the SSPS, version 12.01 [30].

## Results

The sociodemographic characteristics of the patients and healthy control subjects are shown in table 1. The PG patients showed different alterations at the WCST, as compared with healthy control subjects, in particular, perseverant errors, failure to maintain the series and difficulty in learning to learn. As far as the perseverant errors were concerned, the mean score was obtained by eliminating two extreme positive and negative performances, with x = 48 ± 3.2 which corresponds to the mean score of the ratings T = 25+4, while the mean score of the healthy control subjects was significantly lower (x = 18 ± 2.1 and T = 45+9, p < 0.001); according to this score, the patients fell into the moderate performance group. With regard to the failure to maintain a series, the healthy control subjects showed a score (mean ± SD) of 0.87 ± 0.9, while the patients of 1.00 ± 0.55 (p < 0.005), suggestive of a great difficulty in finding alternative methods of problem-solving. The "learning to learn" scores obtained by the patients (-8.66 ± 4) were significantly higher (p < 0.001) than those of the healthy control group (-2.41 ± 5.26), which meant that the patients' efficiency decreased instead of increasing during the consecutive phases of the test, so that they seemed unable to learn from their mistakes. However, the number of categories completed, that is the number of 10 consecutive correct matchings according to the criterion of each category, was not different in the 2 groups (3.7 ± 1.1 and 4.4 ± 2.2, respectively).

**Table 1:** Socio-demographic characteristics of the subjects.

	PG patients Mean (± SD)	HS Mean (± SD)	P value
Age (years)	26.2 (± 4.4)	25.3 (± 5.6)	ns
	**N (%)**	**N (%)**	
Gender			
Men	15 (75)	15 (75)	
Women	5 (25)	5 (25)	ns
Marital Status			
Unmarried (single, separated or divorced)	13 (65)	14 (70)	
Married	7 (35)	6 (30)	ns
Work Status			
Unemployed	11 (55)	8 (40)	
Employed	9 (45)	12 (60)	ns
Education	11 (55)	8 (40)	
Low	9 (45)	12 (60)	ns
High (high school and graduate)			

PG, Pathological Gambler; HS, Healthy control subjects

On the other hand, the score (mean ± SD) of the WMR-S was 97 ± 11 in the patients, not different from that of the healthy subjects (95 ± 10); the same was true for the FAS score (mean ± SD) which was 27 ± 3 in the patients and 28 ± 6 in the control subjects (table 2).

**Table 2:** Scores (mean+SD) of the neuropsychological tests of PG patients (PG) and healthy control subjects (HS).

		PG	HS
WCST	perseverant errors	x = 48 ± 3.2 T = 25 ± 4	x = 18 ± 2.1 T = 45 ± 9*
	failure to maintain	1.00 ± 0.55	0.87 ± 0.9**
	learning to learn	-8.66 ± 4	-2.41 ± 5.26*
	number of categories	3.7 ± 1.1	4.4 ± 2.2
WMR-S		97 ± 11	95 ± 10
FAS		27 ± 3	28 ± 6

* significant: p < 0.001.

** significant: p < 0.005.

WCST = Wisconsin Card Sorting test.

WMR-S = the Wechsler Memory Scale revised.

FAS test = Verbal Associative Fluency Test.

## Discussion

The main bias of our study is that it was carried out in a small sample of PG patients: this was a consequence of the setting in which the patients were selected, namely a public psychiatric department, to which patients with such a disorder are generally sent on account of severe legal problems and/or as a result of strong prompting by their relatives. This might also provide an explanation for the high level of comorbid disorders observed; also, because of the small sample size, we were unable to determine the possible effect of different diagnostic patterns on neuropsychological tests. Nevertheless, our findings may be considered intriguing: in fact, our group of patients affected by PG undergoing a battery of neurological tests, namely the WCST, the WMS-R and the FAS, showed that they had sufficient or normal intellectual, linguistic and visual-spatial abilities. As far as the WCST is concerned, PG patients showed qualitative but not quantitative deficits: in fact, although no differences were found between PG patients and healthy control subjects in the total number of categories completed, different abnormalities were detected at some subscales. As compared with healthy subjects, the thinking of PG patients appeared perseverant, because when they tried to resolve a problem while using an incorrect method, they tended to continue beyond that point at which other subjects would have looked for alternative solutions. A similar behavior has been observed in PG patients at both the card-choosing tests [21] and the GO/NO-GO task [24]. The difficulty that PG patients showed in learning from their mistakes and in re-directing themselves in the appropriate direction represents one of the most characteristic features of patients with alterations of the prefrontal lobe. This aspect has been observed in a significant number of experimental paradigms, in particular, patients with lesions of the prefrontal lobe are sometimes able to identify correct answers, while nevertheless still continuing to produce wrong answers [31-36]. Given the possibility of disproportionate aging of the ventromedial prefrontal areas, which would implicate the decline of decision making with age [37], the sample we selected was homogeneous with regard to that variable.

Our findings are also compatible with other studies reporting worse performances in cognitive "risk-taking" tasks in patients with prefrontal lesions, as compared with healthy control subjects or patients with temporal lobe excision [38]. A link has also been underlined between attention problems or impulsivity in executive functions and minimal brain damage including impairment of the prefrontal lobe [39]; in some cases, the difficulties in controlling behaviour leads such patients to neglect or break the rules of the game [40,41]. Previous studies, although reporting no alterations at the WMS-R and FAS similar to what described by us, could not detect dysfunctions at the WCST, but only at the gambling task in disagreement with our findings [42]. Since the WCST is sensitive to damage to the dorsolateral portion of the prefrontal cortex, as well as to damage to non-prefrontal cortical regions connected to the prefrontal cortex (e.g., parietal cortex) [43,44], despite the preliminary hypothesis of a selective ventromedial prefrontal cortex dysfunction in PG patients [42], our data would suggest a more generalized frontal lobe impairment. This is also supported by a recent study showing alteration of both dorsolateral prefrontal and orbitofrontal cortex in PG [45]. However, it is still unclear whether the observed frontal lobe abnormalities should be considered a primary phenomenon linked to the aetiology of PG, or secondary to some symptomatological features, or to the comorbid psychopathological conditions.

## Conclusion

Taken together, our results would seem to confirm an altered functioning of the prefrontal areas which would determine a cognitive rigidity that might be expressed through altered executive functions and the decision making process; this could represent a factor of vulnerability to the development of impulsive and/or compulsive behaviours, such as those typical of PG. It would be interesting to replicate these findings in patients suffering from PG alone, or with distinct comorbidity patterns.

## Abbreviations

Pathological gambling (PG); Wisconsin Card Sorting Test (WCST); Wechsler Memory Scale revised (WMS-R); Verbal Associative Fluency Test (FAS); Diagnostic and Statistical Manual of Mental Disorders (DSM); obsessive-compulsive disorder (OCD); Yale Brown for Obsessive Compulsive Disorder modified for PG (Y-BOCS-PG).

## Competing interests

The author(s) declare that they have no competing interests.

## Authors' contributions

DM conceived of the study and participated in its design and coordination. MCDO and GC participated in the design and organization of the study and gave the informations about the new reported case. LV, FM, FG and EDN partecipated in the review of the literature and drafted the manuscript. All authors read and approved the final manuscript.
